# Jianpi Yangzheng Xiaozheng granule induced ferroptosis to suppress gastric cancer progression through reprogramming lipid metabolism via SCD1/Wnt/β-catenin axis

**DOI:** 10.3389/fmolb.2025.1523494

**Published:** 2025-02-25

**Authors:** Xiangyang Wang, Jingxiao Li, Rong Qin, Yi Yin, Jiepin Li, Sitian Lin, Xi Zou

**Affiliations:** ^1^ Department of Oncology, Affiliated Hospital of Nanjing University of Chinese Medicine, Jiangsu Province Hospital of Chinese Medicine, Nanjing, Jiangsu, China; ^2^ No. 1 Clinical Medical College, Nanjing University of Chinese Medicine, Nanjing, Jiangsu, China; ^3^ Department of Medical Oncology, Jiangsu University Affiliated People’s Hospital, Zhenjiang, Jiangsu, China; ^4^ Zhenjiang Clinical Medical College of Nanjing Medical University, Zhenjiang, Jiangsu, China

**Keywords:** traditional Chinese medicine, poorly cohesive carcinoma, ferroptosis, lipid metabolism, network pharmacology

## Abstract

The incidence of Poorly cohesive carcinoma (PCC) has steadily risen in recent years, posing a significant clinical challenge. To reveal the anti-tumor effects of Jianpi Yangzheng Xiaozheng granule (JPYZXZ) in PCC, an initial investigation was performed using CCK-8, colony formation, scratch, and transwell assays. This was followed by network pharmacology studies to gain a deeper understanding of JPYZXZ’s impact on gastric cancer (GC). Then reactive oxygen species (ROS), Fe^2+^, malondialdehyde (MDA), glutathione (GSH), Oil Red O staining, BODIPY493/503, triglyceride (TG), and cholesterol (TC) assay kits and western blot (Wb) analysis were applied to exam the regulatory effects of JPYZXZ on ferroptosis and lipid metabolism. Additionally, molecular docking studies and Wb analysis were used to further investigate the mechanisms of JPYZXZ on PCC. Finally, *in vivo* animal studies were conducted. The results show that JPYZXZ can inhibit the proliferation and migration of PCC cell. It increases the levels of ROS, Fe^2+^, MDA, while declining the content of GSH, TC, TG, and lipid droplet accumulation within cellular compartments. Wb indicates that JPYZXZ can negatively regulate the expression of proteins, including glutathione peroxidase 4 (GPX4), cystine/glutamate antipoter SLC7A11 (xCT), fatty acid synthase (FASN), and acetyl coenzyme A carboxylase 1 (ACC1). Furthermore, ferrostatin-1 (fer-1) is able to reverse the effects of JPYZXZ on the aforementioned markers of ferroptosis and lipid metabolism. Molecular docking analyses reveal that JPYZXZ exhibits a favorable binding affinity towards Stearoyl-Coenzyme A desaturase 1 (SCD1). Mechanism studies demonstrate that JPYZXZ is capable of down-regulating the expressions of proteins like SCD1, β-catenin, GPX4, and xCT, which is analogous to the effects of SCD1 knockdown and the application of SCD1 inhibitor A939572. Nevertheless, when SCD1 is knocked down, JPYZXZ is unable to further downregulate the expressions of these proteins. Animal studies have corroborated the *in vitro* tumor-inhibiting effects of JPYZXZ. Therefore, this study offers the first evidence that JPYZXZ inhibits PCC progression by orchestrating ferroptosis and altering lipid metabolism, mediated by the SCD1/Wnt/β-catenin pathway.

## 1 Introduction

Gastric cancer (GC) is a prevalent malignant neoplasm, with 2020 data indicating that its global incidence and mortality rank fifth and fourth, respectively (Sung et al., 2021). GC poses a substantial health burden in China, with a projected 360,000 newly diagnosed cases and 260,000 fatalities expected in 2022 (Han et al., 2024). Poorly cohesive gastric cancer (PC-GC) is a histological subtype of GC, with an increasing incidence and poor prognosis in recent years (Drubay et al., 2022; Grinlinton et al., 2024). Compared to other GC subtypes, PC-GC characterized by isolated or small clusters of tumor cells is more common in young adults and women (Nakamura et al., 2022). Surgery is the primary treatment for PC-GC, but radical surgery is a major challenge due to its susceptibility to invasion of the submucosa and metastasis to the lymph nodes (Liu Y. et al., 2023). Another issue is that PC-GC is prone to peritoneal metastases and responds poorly to conventional therapies, underscoring the need for enhanced prevention and treatment strategies in this population (Liu et al., 2024b).

The popularity of traditional Chinese medicine (TCM) in clinical settings can be attributed to its unique combination of synergistic ingredients, diverse therapeutic targets, and favorable safety profile. Recent researches have demonstrated that the integration of JPYZXZ, a TCM formula developed by Professor Shen-Lin Liu, with chemotherapy, has shown promise in extending survival rates, mitigating adverse effects, and enhancing overall quality of life for patients with advanced GC (Hou et al., 2021; Pan et al., 2020). Our research has demonstrated that JPYZXZ and its constituent parts exhibit inhibitory effects on GC progression, with potential mechanisms involving the modulation of epithelial-to-mesenchymal transition, macrophage polarization, exosomal PD-L1 expression, lipid metabolic pathways, and gut microbiota composition, as evidenced by both *in vivo* and *in vitro* studies (Chen M. et al., 2023; Chen Y. et al., 2023; Wu et al., 2019; Zhu et al., 2024). However, the efficacy and potential mechanism of JPYZXZ on PC-GC have not yet been explored.

Ferroptosis, a novel form of programmed cell death distinct from apoptosis, pyroptosis, and necroptosis that plays a crucial role in the progression and drug resistance in GC, was first coined by the Stockwell lab in 2012 and is characterized by iron-dependent lipid peroxidation (Li Q. et al., 2024a; Stockwell, 2022; Wu et al., 2024). Cancer cells’ rapid growth and active metabolism require increased iron ions and fatty acid, making them more susceptible to ferroptosis (Bian et al., 2021; Jiang et al., 2021). Some ferroptosis-related biomarker models possess the ability to predict the prognosis of patients with GC (Liu Y. J. et al., 2023c). Therefore, targeted ferroptosis may be a promising strategy in tumor therapy. The active components of JPYZXZ have demonstrated the ability to trigger ferroptosis in certain tumors (Feng et al., 2022; Guo et al., 2024; Tong et al., 2023). However, the study of JPYZXZ to induce ferroptosis in PC-GC has not been carried out.

SCD1, a fatty acid synthetase residing in the endoplasmic reticulum, catalyzes the conversion of saturated fatty acids (SFAs) to monounsaturated fatty acids (MUFAs), which are resistant to peroxidation, thereby inhibiting ferroptosis (Igal, 2024; Sen et al., 2023). The high expression of SCD1 in tumors has a poor prognosis, and the mechanism may be through the regulation of lipid metabolism and ferroptosis, resulting in immunosuppression, chemotherapy insensitivity, and resistance to ferroptotic therapy (Che et al., 2024; Fan et al., 2024; Igal, 2024; Li Q. et al., 2024b). Studies have revealed that modulating the Wnt/β-catenin pathway can affect the malignant biological behaviors of tumors (Tang et al., 2023; Tian et al., 2023). Research has established that SCD1 exerts a pivotal role in regulating the Wnt/β-catenin signaling pathway by synthesizing MUFAs and activating porcupine (El Helou et al., 2017; Rios-Esteves and Resh, 2013). In GC cells, SCD1 has the capability to promote cell proliferation and migration, while also up-regulating the expression of anti-ferroptosis markers xCT and GPX4, which are also closely tied to the Wnt/β-catenin signaling pathway (Wang et al., 2020). While targeting SCD1 as a cancer treatment strategy is promising, the clinical application of current drugs has been hindered by their harmful side effects (Sen et al., 2023).

In this study, we have made a significant discovery that the anti-tumor properties of JPYZXZ on PG-GC are linked to the induction of ferroptosis through the SCD1/Wnt/β-catenin signaling pathway and the inhibition of *de novo* fatty acid synthesis. Moreover, we have identified a novel anti-gastric cancer mechanism of JPYZXZ, offering a fresh perspective that enhances our understanding of the anti-tumor mechanisms of traditional Chinese herbal medicine.

## 2 Materials and methods

### 2.1 Reagents

FerroOrange probes were purchased from Dojindo (Kumamoto, Japan). Reactive oxygen species (ROS) and BODIPY493/503 assay kits were purchased from Beyotime (Shanghai, China). Oil Red O solutions were purchased from Solarbio (Beijing, China). Malondialdehyde (MDA), glutathione (GSH), triglyceride (TG) and cholesterol (TC) assay kits were purchased from Nanjing Jiancheng (Nanjing, China). Glutathione peroxidase 4 (GPX4) monoclonal antibody, SLC7A11/xCT polyclonal antibody, SCD polyclonal antibody, fatty acid synthase (FASN) polyclonal antibody, acetyl coenzyme A carboxylase 1 (ACC1) polyclonal antibody, β-catenin polyclonal antibody, and β-actin monoclonal antibody were purchased from Proteintech Group, Inc. (Wuhan, China). Anti-rabbit and anti-mouse antibodies were sourced from CST (Boston, United States). A939572 and ferrostatin-1 (fer-1) were purchased from MedChemExpress (shanghai, China).

### 2.2 Preparation of JPYZXZ decoctions

The herbal granules of JPYZXZ were purchased from Tianjiang pharmaceutical Co., Ltd., which consist of 60 g Astragalus membranaceus (Fisch.) Bunge (Huang Qi) (catalog number: 21062531), 30 g Codonopsis pilosula (Franch.) Nannf. (Dang Shen) (catalog number: 21080721), 10 g Atractylodes macrocephala Koidz. (Bai Zhu) (catalog number: 21062681), 10 g Angelica sinensis (Oliv.) Diels (Dang Gui) (catalog number: 21082261), 10 g Paeonia lactiflora Pall. (Bai Shao) (catalog number: 21061851), 30 g Sparganium stoloniferum (Graebn.) (San Leng) (catalog number: 21041051), 30 g Curcuma zedoaria (Christm.) Roscoe (E Zhu) (catalog number: 21071571), 10 g Vladimiria souliei (Franch.) (Mu Xiang) (catalog number: 21072701), 10 g Citrus reticulata Blanco (Chen Pi) (catalog number: 21061701), 15 g Scleromitrion diffusum (Wild.) (Bai Hua She She Cao) (catalog number: 21074161), 15 g Salvia chinensis Benth. (Shi Jian Chuan) (catalog number: 21060621) and 3 g Glycyrrhiza uralensis Fisch. (Gan Cao) (catalog number: 21073621). All granules were dissolved in double-distilled water, boiled to concentrate to 1 g/mL, and then filtered through a 0.22 μm filter. The extracts were stored at −20°C until use.

### 2.3 Screening the primary active compounds of JPYZXZ

The active compounds of JPYZXZ were derived from the TCMSP database (https://old.tcmsp-e.com). OB ≥ 30% and DL ≥ 0.05 were applied to screen the active compounds of JPYZXZ in this study (Song et al., 2022).

### 2.4 Collection of targets

The PubChem database (https://pubchem.ncbi.nlm.nih.gov) and the Swiss Target Prediction website (www.swisstargetprediction.ch) were used to forecast all potential targets of the active compounds in JPYZXZ. The relevant targets in GC were obtained using the DisGeNET, GeneCards, and OMIM databases. We searched for targets related to ferroptosis in the FerrDB database (www.zhounan.org/ferrdb/). All potential targets and their corresponding active compounds of JPYZXZ were imported into Cytoscape in order to construct the “JPYZXZ active compound-target” network.

### 2.5 Gene ontology (GO) and kyoto encyclopedia of genes and genomes (KEGG) enrichment analysis

To elucidate the molecular mechanisms underlying JPYZXZ-induced ferroptosis in GC, we utilized the Metascape online tool (https://metascape.org/gp/index.html) to perform GO and KEGG enrichment analyses on the identified targets. The analysis generated results across three categories: Biological Process (BP), Cellular Component (CC), and Molecular Function (MF). The enrichment analysis was executed with the following settings: minimum overlap of 3, p-value cutoff of 0.01, and minimum enrichment of 1.5. The top 20 results were graphically represented in bar charts, providing a visual representation of the enriched terms.

### 2.6 Molecular docking

The primary active compounds of JPYZXZ were identified and their mol2 format was obtained from the TCMSP database. Additionally, the X-ray crystal structure files for the SCD1 (4ZYO) protein were acquired from the RCSB PDB (http://www.rcsb.org/). PyMOL 3.0.0 was used for protein preparation, removing water molecules and heteroatoms, and saving the file in. pdb format. AutoDock 4.2.6 and PyMOL 2.5.2 software were utilized for conducting docking studies between compounds and SCD1 proteins. The grid box function of AutoDock Tools was utilized to define a specific pocket for the active ingredients of compounds binding to the proteins. Following this, molecular docking analyses were carried out and the findings were visualized using PyMOL.

### 2.7 Cell culture

The MKN-45 cell line was procured from the Japanese Collection of Research Bioresources Cell Bank, whereas the HGC-27 cell line and human gastric epithelial cell GES-1 were sourced from the Chinese Academy of Sciences in Shanghai, China. The cells were propagated in a nutrient-rich RPMI-1640 medium (Gibco, United States) augmented with 10% fetal bovine serum (EVERY GREEN, China) and 1% penicillin-streptomycin solution (Gibco, United States). The cells were then maintained in a controlled environment at 37°C with a 5% CO_2_ atmosphere, fostering optimal growth conditions.

### 2.8 CCK-8 assay

Briefly, 5 × 10^3^ cells were plated in each well of a 96-well plate and incubated at 37°C with 5% CO_2_ for 24 h. The cells were then exposed to varying concentrations of JPYZXZ (0, 1, 2, 4, 8, 16 and 32 mg/mL) for 24 h or 48 h. Afterward, 10 μL of CCK-8 working solution (Vazyme, China) was added to a 96-well plate and allowed to react for 2 h. The absorbance of the samples at OD 450 nm was subsequently quantified using the Bio Tek 800 TS (Agilent, United States). The formula for calculating cell viability is expressed as follows (experimental group - blank group)/(control group - blank group) × 100%.

### 2.9 Colony formation assay

HGC-27 and MKN-45 cells were seeded at a concentration of 500 cells per well in 6-well plates and exposed to various doses of JPYZXZ (0, 2, 4, and 8 mg/mL) in the culture medium. After a 2-week incubation period, the cells were fixed with a 4% paraformaldehyde solution (1 mL) for 15 min, followed by staining with a 0.1% crystal violet solution (500 μL) for 20 min. Subsequently, high-resolution images of each well were captured, and colonies comprising more than 50 cells were subjected to quantitative analysis.

### 2.10 Wound-healing assay

The impact of JPYZXZ on the migratory capacity of GC cells was assessed through a wound-healing experiment. HGC-27 and MKN-45 cells were seeded at a density of 1 × 10^6^ cells/well in 6-well plates and allowed to reach 95% confluency. Subsequently, a 10 μL pipette tip was used to create uniform scratches, followed by the addition of serum-free medium containing JPYZXZ (0, 2, 4, and 8 mg/mL) to each well. The progress of wound closure was monitored at 0, 24, and 48 h intervals using a microscope (Olympus DP74, Japan) at a magnification of ×100, and the scratch area was assessed using ImageJ software.

### 2.11 Transwell assay

Approximately 5 × 10^4^ cells were seeded into the upper transwell chambers, which contained 200 μL of serum-free medium supplemented with various concentrations of JPYZXZ (0, 2, 4, and 8 mg/mL). The lower compartment was filled with 500 μL of medium containing 10% fetal bovine serum, serving as a chemoattractant. After a 24-h coculture period, the chambers were fixed with 4% paraformaldehyde for 15 min and then stained with 0.1% crystal violet. An Olympus DP74 microscope (Japan) was used to capture images of the cells that had migrated into the lower chambers. Subsequently, ImageJ software was employed to quantify the total number of cells present in the images.

### 2.12 ROS levels determination

Intracellular levels of ROS were assessed using a ROS Assay Kit. HGC-27 and MKN-45 cells were inoculated into 12-well plates at a density of 1 × 10^5^ cells/well according to the manufacturer’s instructions. Thereafter, GC cells were cultured with JPYZXZ (0, 2, 4, and 8 mg/mL) or fer-1 (2 μM) for 24 h. Afterwards, the GC cells were rinsed with serum-free medium and then incubated in a dark environment with 10 μM DCFH-DA for 20 min. Subsequently, intracellular ROS levels were assessed using a fluorescence microscope (Olympus DP74, Japan) and ImageJ software.

### 2.13 Fe^2+^ levels detection

FerroOrange was utilized for the quantification of intracellular Fe^2+^ concentrations in GC cells. Following the manufacturer’s instructions, HGC-27 and MKN-45 cells were seeded at a density of 1 × 10^5^ cells/well in 12-well plates. After adherence, the cells were treated with JPYZXZ (0, 2, 4 and 8 mg/mL) or fer-1 (2 μM) for 24 h, followed by three washes with serum-free medium. Subsequently, the cells were incubated with a working solution of FerroOrange at a concentration of 1 μM in a light-free environment for 30 min. Finally, the fluorescence intensity was measured using a fluorescence microscope (Olympus DP74, Japan) and ImageJ software.

### 2.14 MDA, GSH, TC and TG levels measurement

MDA, GSH, TC and TG levels in GC cells were assessed using corresponding assay kits. HGC-27 and MKN-45 cells were seeded at a density of 1 × 10^6^ cells/well in 3 cm plates. Subsequently, varying concentrations of JPYZXZ (0, 2, 4, and 8 mg/mL) or fer-1 (2 μM) were administered to the cells for 24 h. Following this, the cells were harvested and lysed using ultrasonication. The resulting cell lysates were then treated with appropriate working solutions and MDA, GSH, TC and TG levels were determined using a Bio Tek 800 TS (Agilent, United States).

### 2.15 Lipid staining assay

Oil Red O and BODIPY493/503 staining kits were utilized for the evaluation of neutral lipid content in GC Cells. HGC-27 and MKN45 cells, treated with JPYZXZ (0, 2, 4, and 8 mg/mL) or fer-1 (2 μM), were fixed and stained with the respective work solutions. Subsequently, neutral lipids were determined using a light microscope (Olympus DP74, Japan) and ImageJ software.

### 2.16 Wb assay

Wb assay was performed to assess the protein expression levels in GC cells. The pre-treated cells were lysed using RIPA buffer containing PMFS, phosphatase and protease inhibitors. Subsequently, the protein samples were quantified using a BCA Kit and denatured by heating in boiling water for 10 min. Each sample was then separated on 10% SDS-PAGE gels and transferred onto a PVDF membrane using the wet transfer method. Following blocking with a blocking buffer at room temperature for 15 min, the membranes were immunoblotted overnight at 4°C with primary antibodies targeting SCD1, ACC1, FASN, β-catenin, GPX4, and xCT. After washing three times for 10 min each with TBST, the membranes were incubated with corresponding secondary antibodies for 2 h. Subsequently, the protein signals were detected using the LI-COR 2802 imaging system (LI-COR, United States). Image Studio software was utilized to quantify the protein expression levels.

### 2.17 Gene knockdown

Two small interfering RNAs (siRNAs) targeting SCD1 or a negative control siRNA were designed by Morzan Biotech (Shanghai, China) (Supplementary Table S1). Transfection was performed using Lipofectamine 3000 (Invitrogen, California, United States) according to the manufacturer’s protocol. The transfected cells were cultured for an additional 24 h. Subsequently, the medium was replaced with serum containing JPYZXZ (0 or 8 mg/mL) for both the control and experimental groups. After a further incubation period of 24 h, the cells were harvested for subsequent analyses.

### 2.18 Tumor xenograft experiment

The animal experiments were performed in accordance with relevant guidelines and regulations, which strictly adhering to the ARRIVE guidelines. The ethical approval for the animal study was granted by the Institutional Animal Care and Use Committee of the Affiliated Hospital of Nanjing University of Traditional Chinese Medicine (Approval ID: 2022DW-44-02). Four-week-old male BALB/c nude mice, weighing between 18-20 g, were procured from Beijing Vital River Laboratory Animal Technology Co., Ltd., a reputable supplier in Beijing, China. Each mouse received an injection of 4 × 10^6^ MKN45 cells in the right axilla. Once subcutaneous tumors developed, the mice were randomly assigned to one of five groups: control group (0.2 mL saline administered by gavage daily), JPYZXZ-L group (15 g/kg administered by gavage daily), JPYZXZ-M group (30 g/kg administered by gavage daily), JPYZXZ-H group (45 g/kg administered by gavage daily), and 5-fluorouracil (5-Fu) group (25 mg/kg, 0.02 mL/g administered by intraperitoneal injection every other day). After 14 consecutive days of intervention, the mice were euthanized by cervical dislocation after pentobarbital sodium anesthesia and their tumors were collected for subsequent experiments.

### 2.19 Hematoxylin-eosin (HE) staining

The tissues were embedded in paraffin, sectioned, and then subjected to deparaffinization using xylene and ethanol. Next, the sections underwent hematoxylin staining for 3 min, followed by washing with water, dehydration with alcohol, and staining with eosin solution for 10 s. Afterward, the sections were dehydrated and made transparent using anhydrous ethanol and xylene. Finally, the stained sections were examined and imaged under a microscope for further analysis.

### 2.20 Immunohistochemistry (IHC) assay

Paraffin-embedded tumor specimens were subjected to sectioning and deparaffinization through a series of solvents, including xylene, ethanol, and distilled water. Subsequent antigen retrieval was performed utilizing a microwave oven. To prevent endogenous peroxidase interference, the sections were treated with a 3% hydrogen peroxide solution, followed by a 30-min incubation with 3% bovine serum albumin at ambient temperature. Primary antibodies against SCD1, xCT, GPX4, FASN, ACC1, and β-catenin were added and the sections were incubated overnight at 4°C. Subsequently, HRP-labeled secondary antibody was applied for 50 min at room temperature. DAB chromogen solution was used to visualize the staining while hematoxylin re-stained the nuclei. Dehydration and sealing of the slides preceded imaging under a microscope.

### 2.21 Statistical analysis

GraphPad Prism 10.1.2 software was utilized for the analysis of quantitative data. All experimental data were presented as mean ± SD and the differences among groups were assessed using one-way ANOVA. A p value <0.05 was considered to indicate statistical significance.

## 3 Results

### 3.1 JPYZXZ inhibits the proliferation and migration of PC-GC cell

**FIGURE 1:**
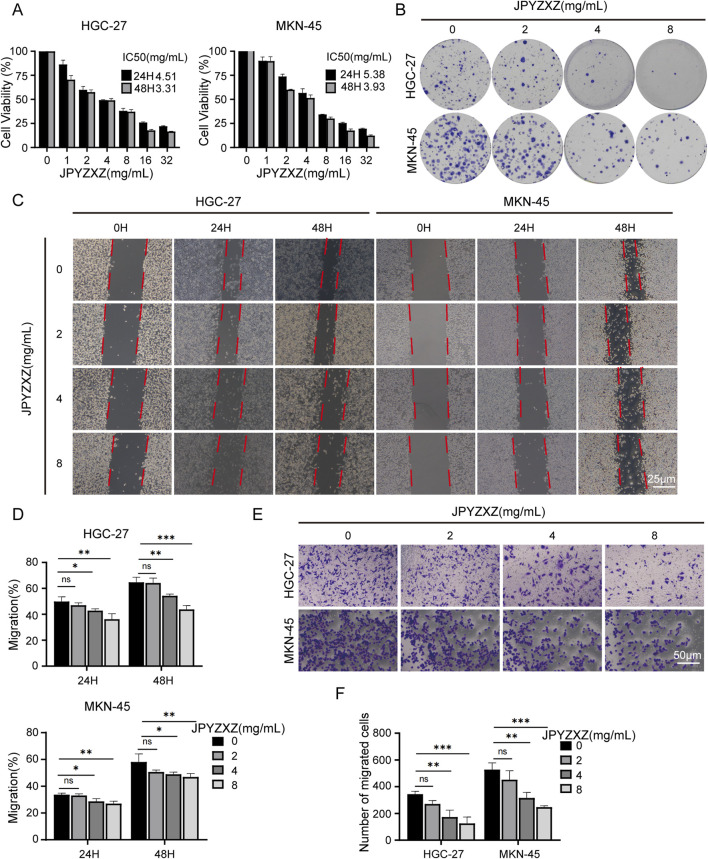
JPYZXZ inhibited the proliferation and migration of HGC-27 and MKN-45 cells. **(A)** The CCK-8 assay was employed to determine the IC50 value of GC cells following treatment with JPYZXZ for 24 and 48 h. **(B)** After exposing GC cells to concentrations of 0, 2, 4, and 8 mg/mL of JPYZXZ for a 24-h period, the cells were cultured for an additional 14 days, and then the colonies of the GC cells were assessed. **(C, D)** The wound-healing assay was employed to assess the impact of JPYZXZ on the migratory capacity of GC cells following treatments at 0 h, 24 h, and 48 h. **(E, F)** The impact of JPYZXZ on the migratory ability of GC cells was assessed via a transwell assay following 24-h exposure. Data are expressed as mean ± SD, n = 3. *p < 0.05, **p < 0.01, ***p < 0.001.

The CCK-8 assay was used to assess the inhibitory effect of JPYZXZ on HGC-27 and MKN-45 cells. Following exposure to various concentrations of JPYZXZ, the IC50 values for HGC-27 and MKN-45 cells were determined as 4.51 and 5.38 mg/mL at 24 h, and 3.31 and 3.93 mg/mL at 48 h, respectively (Figure 1A). Additionally, JPYZXZ exhibited a significant dose- and time-dependent inhibition of GC cells viability, but no obvious toxicity to GES-1 cells (Supplementary Figure S1A). Therefore, concentrations of JPYZXZ (0, 2, 4, and 8 mg/mL) were selected for subsequent experiments. The colony formation assay was employed to further assess the impact of JPYZXZ on GC cells growth, revealing a significant decrease in the number of colonies with increasing concentration of JPYZXZ (Figure 1B). Furthermore, wound healing assay and transwell assay demonstrated that JPYZXZ significantly suppressed the migration of HGC-27 and MKN-45 cells at concentrations of 4 and 8 mg/mL but not at 2 mg/mL (Figures 1C–F). Therefore, we can conclude that JPYZXZ possesses the capability to inhibit the proliferation and migration of PC-GC cell.

### 3.2 Network pharmacology analysis

**FIGURE 2:**
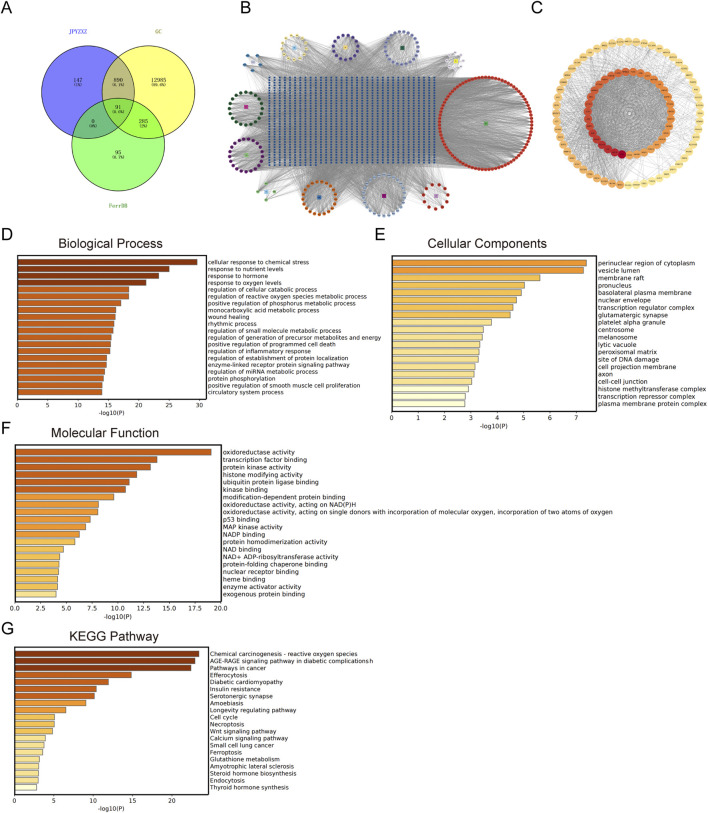
The network pharmacology of JPYZXZ in the management of GC. **(A)** Venn diagram illustrating the overlap of JPYZXZ targets, GC therapeutic targets, and FerrDB database. **(B)** JPYZXZ compound-target network. The square symbolizes the herb, the regular octagon represents the active ingredient of JPYZXZ, and the blue square signifies the target. **(C)** The protein-protein interaction network of the 91 overlapping targets. **(D)** Biological process of GO enrichment analysis on 91 overlapping targets. **(E)** Cellular components of GO enrichment analysis on 91 overlapping targets. **(F)** Molecular function of GO enrichment analysis on 91 overlapping targets. **(G)** KEGG enrichment analysis on 91 overlapping targets.

Previous study has identified the components of JPYZXZ using UPLC-OrbitRAP-MS (Chen M. et al., 2023). We obtained 253 active components of JPYZXZ from the TCMSP database, including 21 Huangqi, 21 Dangshen, 13 Baizhu, 15 Baishao, 20 Danggui, 26 Muxiang, 11 Chenpi, 8 Sanleng, 23 Ezhu, 5 Baihuasheshecao, 4 Shijianchuan, and 86 Gancao. Additionally, a total of 14,251 targets related to GC, as well as 471 targets related to ferroptosis and 1,132 targets related to JPYZXZ were obtained from DisGeNET, GeneCards, OMIM, FerrDB databases and Swiss Target Prediction website. Subsequently, Venny website was utilized to identify 91 overlapped targets (Figure 2A). Following this, JPYZXZ compound-target network and PPI network were constructed (Figures 2B, C). Finally, enrichment analysis of GO/KEGG pathway was performed using Metascape database, and top 20 categories for each were presented. The GO enrichment analysis revealed three primary categories: BP, CC, and MF. Within BP, the dominant themes included cellular responses to oxidative stress, reactive oxygen species, and oxygen levels, as well as metabolic pathways involving unsaturated fatty acids, fatty acid biosynthesis, and lipid oxidation (Figure 2D). Meanwhile, CC were linked to the perinuclear region of cytoplasm, vesicle lumen, membrane raft, and endoplasmic reticulum lumen (Figure 2E). MF were characterized by enzyme activities related to oxidoreduction, fatty acid binding, iron ion interactions, and protein kinase functions (Figure 2F). KEGG pathway analysis revealed involvement in chemical carcinogenesis-reactive oxygen species pathways in cancer, Wnt signaling pathway, ferroptosis, glutathione metabolism, lipid and atherosclerosis (Figure 2G). Both JPYZXZ and Yiqi Huayu decoction are modified versions of the ancient formula “Gui Shao Liu Jun Zi”, which has been used for gastrointestinal diseases for thousands of years. Previous studies have found that Yiqi Huayu plays a crucial role in preventing recurrence and reducing cisplatin resistance in the treatment of GC, both of which are associated with the induction of ferroptosis (Huang et al., 2024; Song et al., 2022). In this study, network pharmacology analysis suggested that the mechanism of JPYZXZ in GC treatment may be associated with ferroptosis, lipid metabolism and Wnt signaling pathway.

### 3.3 JPYZXZ facilitates ferroptosis of PC-GC cell

**FIGURE 3:**
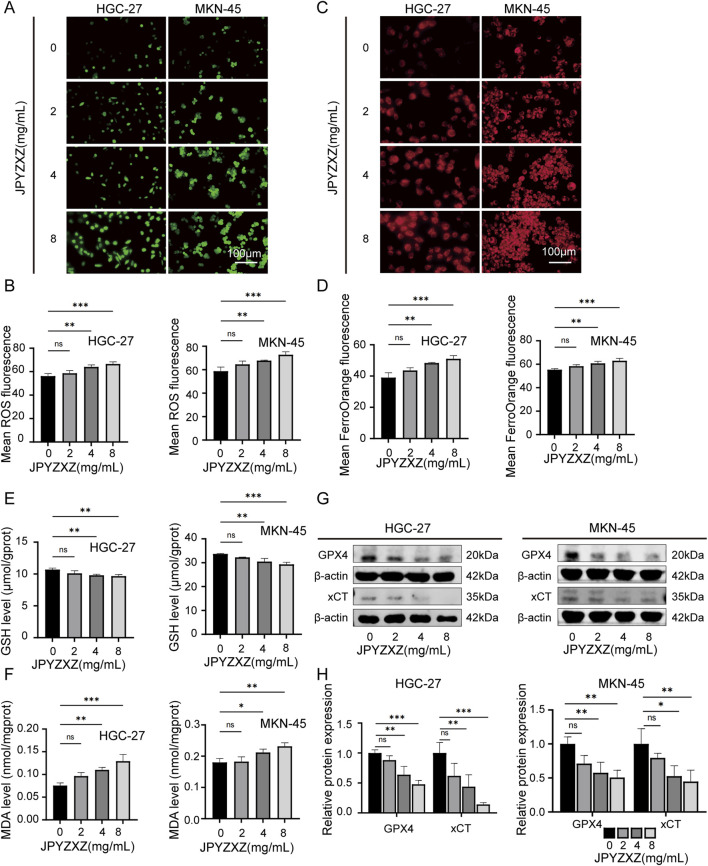
JPYZXZ induced ferroptosis in HGC-27 and MKN-45 cells. **(A, B)** The ROS Assay Kit was utilized to quantify the ROS levels in GC cells treated with 0, 2, 4, and 8 mg/mL of JPYZXZ for 24 h **(C, D)** FerroOrange fluorescent probes were employed for the detection of iron ion levels in GC cells following JPYZXZ treatment. **(E)** The GSH kit was utilized for quantifying the GSH levels in GC cells. **(F)** The MDA kit was utilized for the quantification of MDA levels in GC cells. **(G, H)** The Western blot analysis was employed for the detection of GPX4 and xCT in GC cells. Data are expressed as mean ± SD, n = 3. *p < 0.05, **p < 0.01, ***p < 0.001.

In this study, several key experiments related to ferroptosis were conducted to investigate whether JPYZXZ induces ferroptosis in PC-GC cells. The results showed that JPYZXZ increased intracellular ROS and Fe^2+^ levels in HGC-27 and MKN-45 cells in a dose-dependent manner (Figures 3A–D). Additionally, GSH levels decreased (Figure 3E), while MDA levels increased in both cell lines when treated with 4 and 8 mg/mL of JPYZXZ (Figure 3F). Wb assays revealed that protein levels of GPX4 and xCT significantly decreased in HGC-27 and MKN-45 cells after exposure to 4 and 8 mg/mL of JPYZXZ (Figures 3G, H). However, no significant changes were observed when cells were treated with 2 mg/mL of JPYZXZ.

**FIGURE 4:**
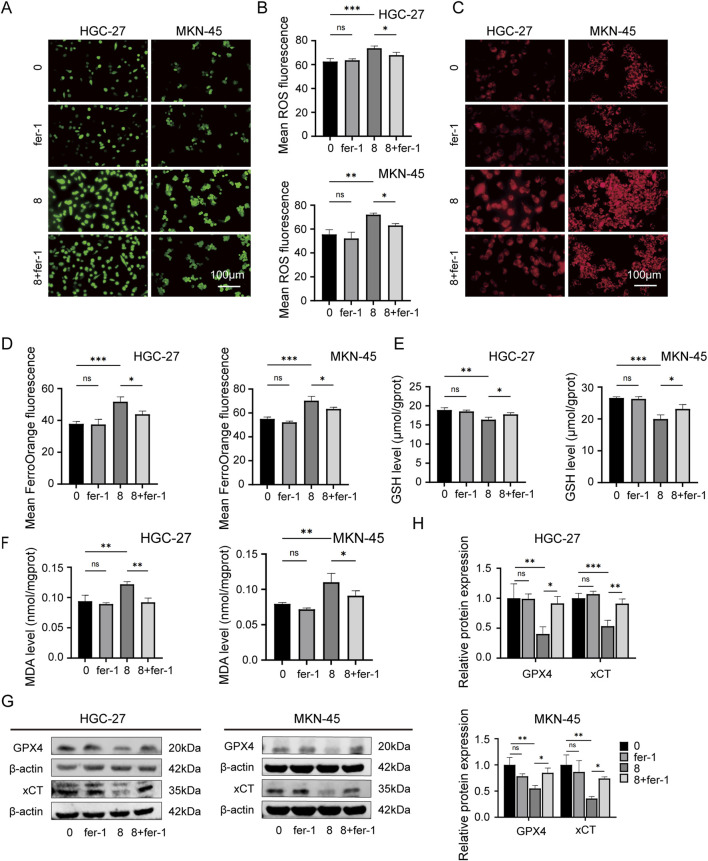
Rescue experiment of ferroptosis induced by JPYZXZ in HGC-27 and MKN-45 cells. **(A, B)** After treatment with 8 mg/mL of JPYZXZ, fer-1 (2 μM), or a combination of both, the levels of ROS in GC cells were measured. **(C, D)** FerroOrange fluorescent probes were used to detect levels of iron ions in GC cells after treatment with JPYZXZ or fer-1. **(E)** The GSH kit was employed to measure the levels of GSH in GC cells. **(F)** The MDA kit was employed to quantify the levels of MDA in GC cells. **(G, H)** The Western blot analysis was utilized for the quantification of GPX4 and xCT in GC cells. Data are expressed as mean ± SD, n = 3. *p < 0.05, **p < 0.01, ***p < 0.001.

Then the rescue experiments were conducted. Fer-1, a widely used inhibitor of ferroptosis, was utilized as a positive control drug for ferroptosis. HGC-27 and MKN-45 cells were treated with JPYZXZ (0 mg/mL), JPYZXZ (8 mg/mL), fer-1 (2 μM), and JPYZXZ + fer-1 (8 mg/mL JPYZXZ +2 μM fer-1) for 24 h. The results showed that fer-1 was able to reverse JPYZXZ-induced elevation of ROS (Figures 4A, B), Fe^2+^ (Figures 4C, D), and MDA (Figure 4F), as well as the reduction of GSH in GC cells (Figure 4E). Wb experiments also confirmed these results, showing that the decrease of GPX4 and xCT in GC cells induced by JPYZXZ could be rescued with fer-1 (Figures 4G, H). Therefore, these observations indicated that JPYZXZ could induce ferroptosis in PC-GC cell.

### 3.4 JPYZXZ-induced ferroptosis is connected with lipid metabolism in PC-GC cell

**FIGURE 5:**
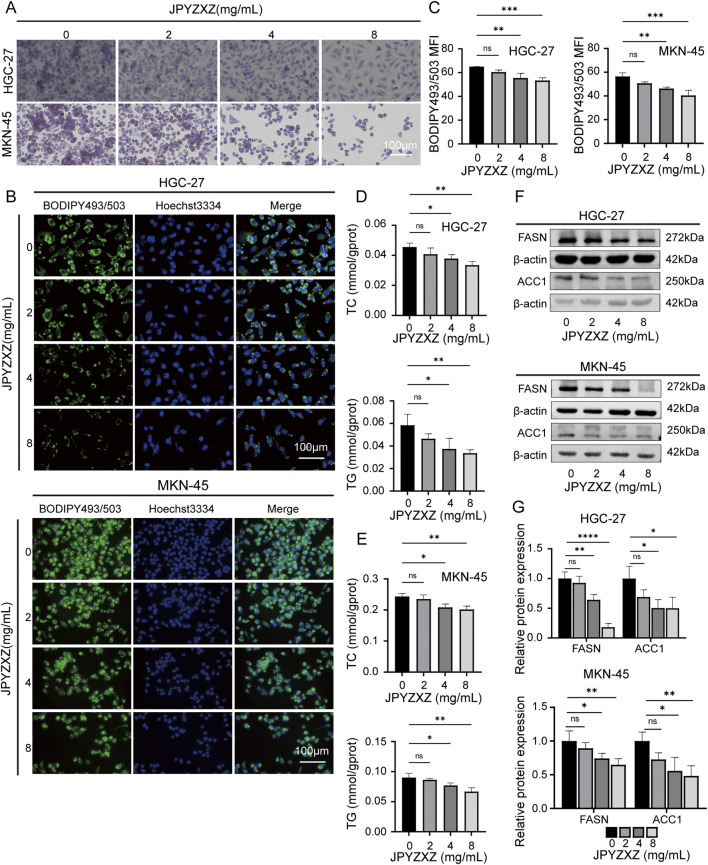
JPYZXZ suppressed lipid accumulation in HGC-27 and MKN-45 cells. **(A)** Oil Red O staining was used to assess the impact of JPYZXZ (0, 2, 4, and 8 mg/mL) on lipid droplet accumulation in GC cells. **(B, C)** BODIPY493/503 assessed the suppression of neutral lipid accumulation in GC cells by JPYZXZ. **(D, E)** The inhibitory effect of JPYZXZ on the content of TC and TG in GC cells was assessed using TC, TG assay kit. **(F, G)** The Western blot assay was employed to assess the suppression of FASN and ACC1 protein expression in GC cells by JPYZXZ. Data are expressed as mean ± SD, n = 3. *p < 0.05, **p < 0.01, ***p < 0.001, ****p < 0.0001.

To investigate the effect of JPYZXZ on lipid metabolism, we evaluated the levels of neutral lipids in GC cells. Initially, HGC-27 and MKN-45 cells were treated with varying concentrations of JPYZXZ (0, 2, 4, and 8 mg/mL) for 24 h. Oil Red O and BODIPY 493/503 assays were utilized to quantify lipid droplet content, revealing a dose-dependent decrease in lipid droplet formation in GC cells following JPYZXZ treatment (Figures 5A–C). Additionally, JPYZXZ significantly inhibited the levels of TC and TG in GC cells (Figures 5D, E). Western blot analysis revealed a significant decrease in the expression of FASN and ACC1 proteins, which are crucial enzymes involved in lipid metabolism in GC cells following treatment with JPYZXZ (Figures 5F, G). Notably, no significant changes were observed at a lower concentration of JPYZXZ (2 mg/mL).

**FIGURE 6:**
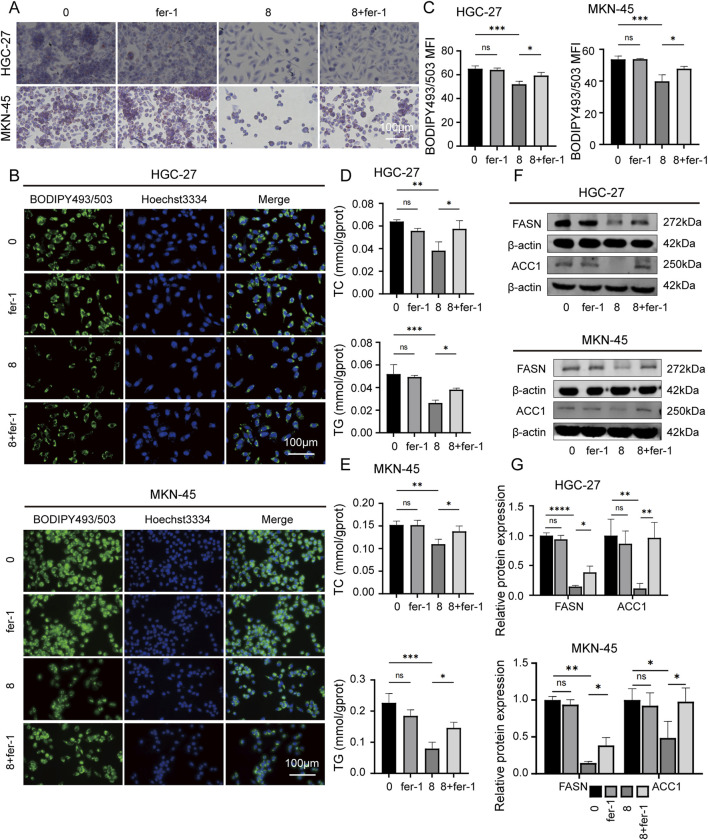
JPYZXZ-induced ferroptosis is associated with the regulation of lipid metabolism in HGC-27 and MKN-45 cells. **(A)** The alterations in lipid droplets of the GC cells were evaluated using Oil Red O staining after treating with JPYZXZ (8 mg/mL) or fer-1 (2 μM) for 24 h. **(B, C)** BODIPY493/503 was utilized to evaluate the accumulation of neutral lipids in GC cells by JPYZXZ or fer-1. **(D, E)** The TC and TG assay kits were used to assess the content of TC and TG in GC cells after treatment with JPYZXZ or fer-1. **(F, G)** The Western blot assay was utilized to evaluate the expression of FASN and ACC1 in GC cells subsequent to treatment with JPYZXZ or fer-1. Data are expressed as mean ± SD, n = 3. *p < 0.05, **p < 0.01, ***p < 0.001, ****p < 0.0001.

To further reveal the potential connection between JPYZXZ-induced ferroptosis and lipid metabolism, the other assay was conducted using four groups: JPYZXZ (0 mg/mL), JPYZXZ (8 mg/mL), fer-1 (2 μM), and combination treatment with both substances. The results indicated that fer-1 was able to reverse the decrease in lipid droplet content (Figures 6A–C), as well as TC and TG levels (Figures 6D, E), while also restoring FASN and ACC1 expression caused by high-dose JPYZXZ treatment (Figures 6F, G). These findings suggest that JPYZXZ-induced ferroptosis is linked to alterations in lipid metabolism within PC-GC cell.

### 3.5 JPYZXZ-induced ferroptosis in PC-GC cell through the SCD1/Wnt/β-catenin axis

**FIGURE 7:**
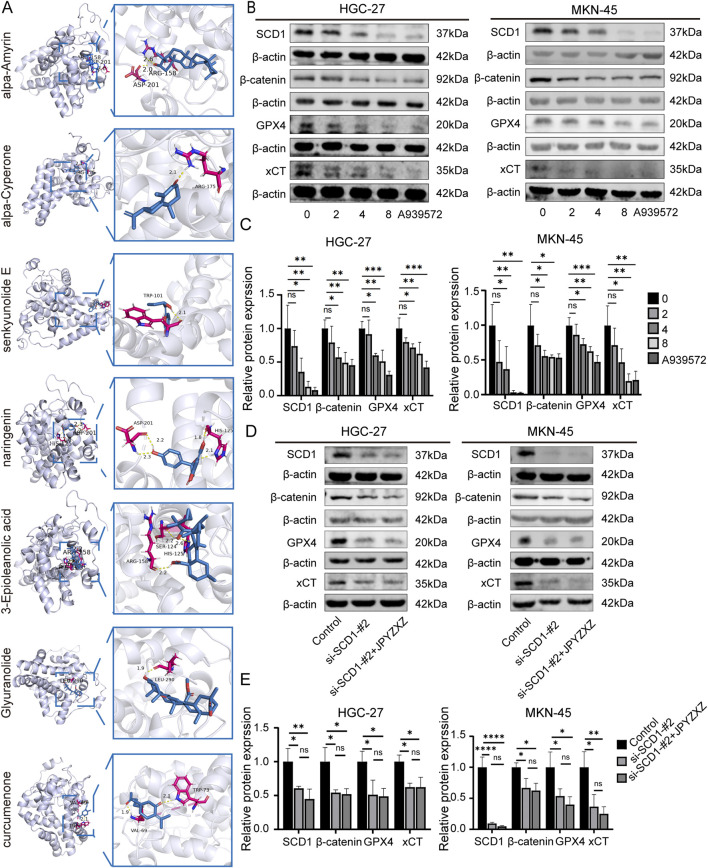
JPYZXZ-induced ferroptosis is related to the SCD1/Wnt/β-catenin signaling axis in HGC-27 and MKN-45 cells. **(A)** Visualization of the molecular docking analysis conducted between the bioactive constituents of JPYZXZ and SCD1. **(B, C)** Western blot was used to quantify the expression of SCD1, β-catenin, GPX4, and xCT in GC cells after treatment with JPYZXZ (0, 2, 4, and 8 mg/mL) and A939572 (5 μM) for 24 h **(D, E)** Western blot was used to quantify the expression of SCD1, β-catenin, GPX4, and xCT in SCD1 knockdown GC cells after treatment with JPYZXZ (0 and 8 mg/mL) for 24 h. Data are expressed as mean ± SD, n = 3. *p < 0.05, **p < 0.01, ***p < 0.001, ****p < 0.0001.

Among the 91 overlapping genes, SCD1 stands out as a key rate-limiting enzyme involved in *de novo* fatty acid synthesis, and its crucial role as a regulator of ferroptosis has been recognized (Sen et al., 2023). Subsequently, 12 compounds from JPYZXZ were selected based on their herbal degree and subjected to molecular docking studies with SCD1 (4ZYO). The results revealed that seven compounds exhibited strong binding affinity: Alpha-Amyrin (Baizhu) = −8.04, 3-Epioleanolic acid (Baihuasheshecao) = −7.95, Glyuranolide (Gancao) = −6.15, alpha-Cyperone (Muxiang) = −6.07, naringenin (Chenpi) = −5.54, Curcumenone (Ezhu) = −5.03 and Senkyunolide E (Danggui) = −5.03 (Figure 7A).

The Wnt/β-catenin signaling pathway is extensively researched and implicated in various aspects of tumorigenesis across multiple cancer types (Zhou et al., 2022). Activation of the Wnt/β-catenin pathway has been shown to inhibit ferroptosis in GC cells by reducing ROS production (Wang et al., 2022). SCD1 has been found to modulate the Wnt/β-catenin pathway in different cancers, leading to upregulation of β-catenin expression in GC cells (Wang et al., 2020). To investigate JPYZXZ-induced ferroptosis via the SCD1/Wnt/β-catenin axis in GC cells, we utilized the SCD1 inhibitor A939572, a small molecule compound known for its ability to inhibit migration, invasion, self-renewal, and chemotherapy resistance of GC cells (Gao et al., 2020). Wb experiments were performed after treating HGC-27 and MKN-45 cells with various concentrations of JPYZXZ (0, 2, 4, and 8 mg/mL) and A939572 (5 μM) for 24 h. The results showed a dose-dependent inhibition of SCD1, β-catenin, GPX4, and xCT expression following treatment with JPYZXZ and A939572 (Figures 7B, C). Then, we established the SCD1 knockdown model of GC and verified the knockdown effect by Wb (Supplementary Figures S1B, C). Then, GC cells with SCD1 knockdown were treated with JPYZXZ. The transfection of si-SCD1 decreased the protein of β-catenin, GPX4 and xCT. However, these proteins of SCD1 knockdown cells had no significant reduced after JPYZXZ treated (Figures 7D, E). These findings led to the conclusion that JPYZXZ induces ferroptosis in PC-GC cell through its interaction with the SCD1/Wnt/β-catenin axis.

### 3.6 JPYZXZ suppressed the growth of PC-GC xenograft

**FIGURE 8:**
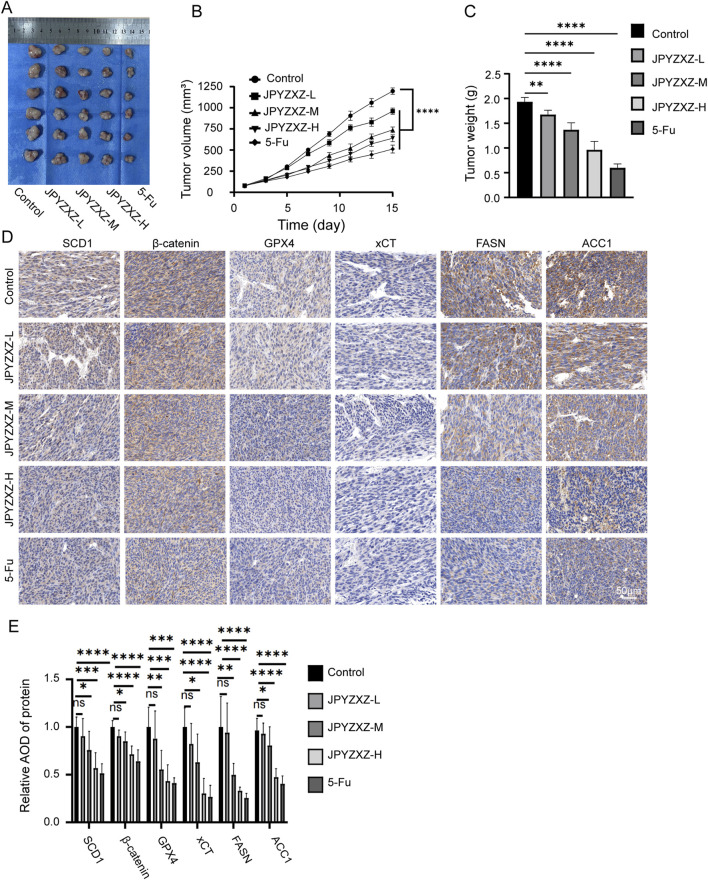
JPYZXZ suppressed the growth of PC-GC xenograft. **(A)** Photographs depicting subcutaneous tumors in nude mice. **(B)** Fluctuations in tumor volume over the course of the experiment. **(C)** The tumor weight on day 15 of treatment. **(D, E)** The IHC analysis was conducted to assess the expressions of SCD1, β-catenin, GPX4, xCT, FASN and ACC1 in subcutaneous tumors of each group. Data are expressed as mean ± SD, n = 6. *p < 0.05, **p < 0.01, ***p < 0.001, ****p < 0.0001.

To verify the potential therapeutic effect of JPYZXZ against PC-GC *in vivo*, a subcutaneous xenograft model was established. The tumor volume and weight were significantly decreased in the 5-fluorouracil (5-Fu) group and JPYZXZ groups compared to the control group (Figures 8A–C). In addition, IHC analysis of tumor sections revealed a dose-dependent reduction in the expression of SCD1, β-catenin, GPX4, xCT, FASN, and ACC1 (Figures 8D, E). Finally, body weight of GC xenograft and HE-stained tissue sections of the heart, lung, liver, and kidney were examined to evaluate the *in vivo* non-toxicity of JPYZXZ (Supplementary Figures S1D,E). Consequently, these findings indicate that JPYZXZ was non-toxic in our PC-GC xenograft model. Hence, we can infer that the effective inhibition of PC-GC growth *in vivo* by JPYZXZ is linked to ferroptosis, lipid metabolism and SCD1/Wnt/β-catenin axis.

## 4 Discussion

The high prevalence of GC in China has placed a substantial burden on both individuals and society. Although screening for GC and eradicating *Helicobacter pylori* have contributed to a decline in its incidence, patients with PCC are still on the rise (Sukri et al., 2020; Yen et al., 2024). PCC is more aggressive and has poorer survival outcomes compared to non-PCC (Koseki et al., 2023). Drug therapy is essential for PCC with the challenges of radical surgery; however, due to its heterogeneity, inefficiency, drug resistance, and side effects remain significant issues that impact patient survival (Baek et al., 2023; Depotte et al., 2024). Thus, the development of safe and efficacious therapeutic agents for the management of PCC is a pressing priority, emphasizing the importance of identifying novel treatments with minimal toxicity and optimal clinical outcomes.

TCM, a distinct and ancient practice with a remarkable history of over 2,000 years of clinical application, has made significant contributions to our health and wellbeing (Luo et al., 2021). Artemisinin, derived from the Chinese herb Artemisia annua and recognized by the WHO as an essential medicine for malaria treatment due to its remarkable efficacy, led Youyou Tu to win the 2015 Nobel Prize in Physiology or Medicine for her discovery (Shi et al., 2022). Consequently, TCM has sparked extensive research interest, including its application in treating cancer. Studies have revealed that both monomeric components and compound formulas of traditional Chinese medicine exert certain auxiliary roles in tumor therapy, including the treatment of GC (Liu B. et al., 2023a; Yu et al., 2023; Zhu et al., 2023). According to TCM theory, most GC patients exhibit blood stasis and weakness in the spleen and stomach. Professor Liu’s creation JPYZXZ has been found to invigorate Qi, strengthen the spleen and remove stasis. Clinical studies have demonstrated its ability to enhance efficiency, reduce toxicity and prolong survival in advanced GC cases (Hou et al., 2021; Pan et al., 2020). While JPYZXZ and its components have shown inhibitory effects on GC progression through *in vitro* and *in vivo* experiments, the efficacy and therapeutic mechanism of JPYZXZ against PC-GC remains unclear (Chen Y. et al., 2023; Wu et al., 2022; Xu et al., 2023). In order to understand the effect of JPYZXZ on PC-GC, MKN-45 derived from signet ring carcinoma was selected for experiments. Initially, CCK-8 and colony experiments revealed that JPYZXZ could inhibit the proliferation of GC cells. Compared with the undifferentiated cell line HGC-27, MKN-45 has a higher IC50, implying that PG-GC exhibits a greater malignancy. Subsequently, wound healing and transwell assays demonstrated a dose-dependent inhibition of GC cells migration by JPYZXZ. These findings are consistent with existing literature, supporting the proposal that JPYZXZ possess the capability to inhibit both proliferation and migration of PC-GC cell.

Ferroptosis is a type of cell death characterized by iron overload, lipid peroxidation, and ROS accumulation. However, cancer cells have developed resistance to ferroptosis by activating antioxidant defenses. Specifically, they use the system xc^−^ to transport cystine into the cell, which is then reduced to cysteine. The cysteine is subsequently combined with glutamate to produce GSH, a crucial antioxidant. GSH is then utilized by the enzyme GPX4 to convert harmful polyunsaturated fatty acid phospholipid (PUFA-PL) hydroperoxides into non-toxic PUFA-PL alcohols, thereby neutralizing the toxic effects of ferroptosis (Stockwell, 2022). Thus, ferroptosis can be inhibited by reducing the contents of intracellular iron ions, lipid peroxides, ROS accumulation and increasing the expression of system xc^−^ and GPX4. Based on the network pharmacology analysis, we speculated that JPYZXZ could induce ferroptosis in GC cells. We utilized ROS and MDA assays to measure the levels of lipid peroxides in GC cells. Our findings revealed that JPYZXZ exhibited a dose-dependent increase in lipid peroxides. Additionally, we employed FerroOrange kit to assess the cellular Fe^2+^, which demonstrated a dose-dependent elevation upon treatment with JPYZXZ. Subsequently, we utilized GSH kit and Wb experiments to evaluate the impact of JPYZXZ on the xCT/GSH/GPX4 antioxidant axis. Our results indicated that JPYZXZ led to a dose-dependent reduction in xCT, GSH and GPX4, thereby attenuating the intracellular antioxidant capacity. Ultimately, we performed rescue experiments using fer-1, which demonstrated that fer-1 successfully restored the elevated levels of lipid peroxides and Fe^2+^ in GC cells treated with JPYZXZ. Additionally, it was observed that fer-1 rescued the downregulation of the xCT/GSH/GPX4 antioxidant axis with the treatment of JPYZXZ. Consequently, it can be inferred that JPYZXZ exert its inhibitory effects on PC-GC progression by inducing ferroptosis.

Lipids play a crucial role in maintaining normal cellular function through the formation of cell membranes, signaling molecules, and provision of energy (Bian et al., 2021). The proliferation of tumor cells necessitates increased lipid involvement, highlighting the significance of lipid metabolism which involves numerous enzymes (Chen M. et al., 2023). The synthesis of fatty acids commences with the catalysis of acetyl-CoA by ACC to yield malonyl-CoA, followed by the catalysis of malonyl-CoA and acetyl-CA by FASN to generate palmitic acid. Subsequently, palmitic acid undergoes catalysis by SCD1, resulting in the production of MUFA (Li et al., 2022). Inhibition of enzymes involved in fatty acid metabolism has been shown to impede the progression of GC (Cui et al., 2022). To investigate the inhibition of JPYZXZ on GC progression is related to the regulation of lipid metabolism, we assessed intracellular TG and TC levels of GC cells. Our findings revealed a dose-dependent reduction in TC and TG contents in GC cells following treatment with JPYZXZ. Furthermore, we used Oil Red O staining and BODIPY493/503 to measure the intracellular lipid droplet content and observed a dose-dependent decrease induced by JPYZXZ. Wb analysis further confirmed that JPYZXZ treatment downregulated fatty acid synthase enzymes FASN and ACC1. We then used ferroptosis inhibitor fer-1, which reversed the JPYZXZ-induced downregulation of TC, TG, lipid droplets, FASN, and ACC1. These findings suggest that JPYZXZ regulates ferroptosis in PC-GC by suppressing lipid metabolism.

Network pharmacology utilizes computational algorithms to construct a network connecting diseases and drugs, thereby elucidating the targets and molecular mechanisms of the active components found in TCM (Liu J. et al., 2024a). We employed a comprehensive approach to identify potential targets of JPYZXZ, utilizing three databases: TCMSP, PubChem, and Swiss Target Prediction. To determine the targets related to GC, we consulted DisGeNET, GeneCards, and OMIM databases. Additionally, we used FerrDB to identify targets associated with ferroptosis. The overlap of these datasets resulted in 91 shared targets, with one of the most significant being SCD1. SCD1 is a rate-limiting enzyme that catalyzes SFAs to produce MUFAs, which displace PUFAs from phospholipids, inhibiting the synthesis of PUFA-PLs and their peroxidation, thus protecting against ferroptosis (Lei et al., 2024). Recent studies have found that SCD1 has the ability to sustain GC malignancy through inhibiting ferroptosis via binding of exo-lncFERO to SCD1 mRNA and recruitment of hnRNPA1, or SQLE/cholesterol/mTOR signaling pathway (Mao et al., 2024; Zhang et al., 2021). Hence, the targeting of SCD1 has the potential to impede the progression of GC and facilitate ferroptosis in GC cells. To investigate the regulatory impact of JPYZXZ on SCD1, we conducted molecular docking analysis, a method capable of simulating the interaction between small molecular compounds and proteins. Through the analysis of binding affinity and sites, we can identify effective compounds that interact with this protein, with a binding energy of < −5 kcal/mol indicating a strong binding affinity (Agu et al., 2023; Song et al., 2022). Among the 12 active ingredients in the JPYZXZ formula, 7 compounds demonstrate a strong binding affinity with SCD1, including Alpha-Amyrin, 3-Epioleanolic acid, Glyuranolide, alpha-Cyperone, naringenin, Curcumenone and Senkyunolide E. Furthermore, several of these compounds have demonstrated efficacy in various cancer types (Li Y. Z. et al., 2024; Neto et al., 2021; Pei et al., 2020). Hence, the suppressive impact of the JPYZXZ prescription on GC may be attributed to its inhibition of SCD1. Subsequently, a KEGG analysis was performed, revealing that the principal pathway implicated in ferroptosis by the JPYZXZ in GC may entail modulation of the Wnt signaling pathway. The Wnt/β-catenin pathway is a canonical component of the Wnt signaling cascade (Zhou et al., 2022). Activation of this pathway results in the accumulation of β-catenin, which subsequently translocates to the nucleus and forms a complex with TCF/LEF to induce the expression of GPX4, thereby suppressing ferroptosis in GC cells (Wang et al., 2022). Consequently, inhibition of the Wnt/β-catenin pathway can trigger ferroptosis in GC. Recent research has shown that SCD1 exerts a significant regulatory influence on the Wnt/β-catenin pathway in GC, contributing to the advancement of this disease (Wang et al., 2020; Wong et al., 2023). Therefore, we postulated that JPYZXZ modulates ferroptosis in GC via the SCD1/Wnt/β-catenin pathway. We utilized the SCD1 inhibitor A939572 and conducted western blot experiments, which revealed that both JPYZXZ and A939572 were capable of suppressing the expression of SCD1, β-catenin, GPX4, and xCT. The reduction of these proteins was also observed in GC cell model with SCD1 knockdown. However, compared with the si-SCD1 group, the protein levels of SCD1, β-catenin, GPX4, and xCT in the si-SCD1 cells subjected to JPYZXZ treatment did not exhibit further decline. Consequently, we concluded that JPYZXZ modulates ferroptosis in PC-GC cell through the SCD1/Wnt/β-catenin pathway.

Subsequently, *in vivo* experiments were conducted utilizing JPYZXZ, revealing a dose-dependent reduction in the volume of subcutaneous tumors in nude mice. Importantly, no significant toxicity of JPYZXZ to mice was observed by body weight monitoring and HE staining of vital organs such as heart, lung, liver and kidney. Furthermore, IHC analysis demonstrated a dose-dependent downregulation of SCD1, GPX4, xCT, FASN, ACC1 and β-catenin expressions. As a result, it is inferred that JPYZXZ may hinder the progression of PC-GC by modulating the SCD1/Wnt/β-catenin axis, inducing ferroptosis, and inhibiting fatty acid synthesis, consistent with findings from cellular experiments.

## 5 Conclusion

**FIGURE 9:**
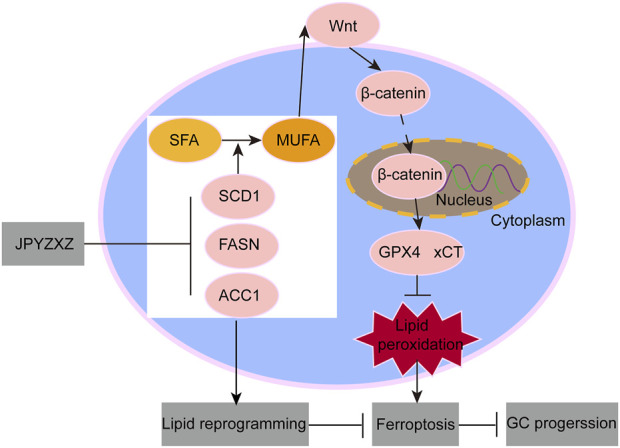
Schematic diagram of JPYZXZ induces ferroptosis suppressing GC progression.

In conclusion, this study has identified a novel regulatory mechanism by which JPYZXZ inhibits the progression of PC-GC. Specifically, JPYZXZ modulates intracellular lipid composition may through the inhibition of key fatty acid synthetases, including SCD1, FASN, and ACC1. Furthermore, JPYZXZ suppresses the antioxidant axis via the SCD1/Wnt/β-catenin pathway, leading to increased peroxide levels, which in turn trigger ferroptosis and ultimately hinder the progression of PC-GC (Figure 9).

## Data Availability

The original contributions presented in the study are included in the article/Supplementary Material, further inquiries can be directed to the corresponding author.
