# Supplementary material for: Jianpi Yangzheng Xiaozheng granule induced ferroptosis to suppress gastric cancer progression through reprogramming lipid metabolism via SCD1/Wnt/β-catenin axis
Source: Front Mol Biosci. 2025 Feb 25;12:1523494. doi: 10.3389/fmolb.2025.1523494 (PMC11893430; doi:10.3389/fmolb.2025.1523494)
Supplement: Supplementary file 1 [file DataSheet1.pdf]

# Supplementary Material

## Supplementary Figures

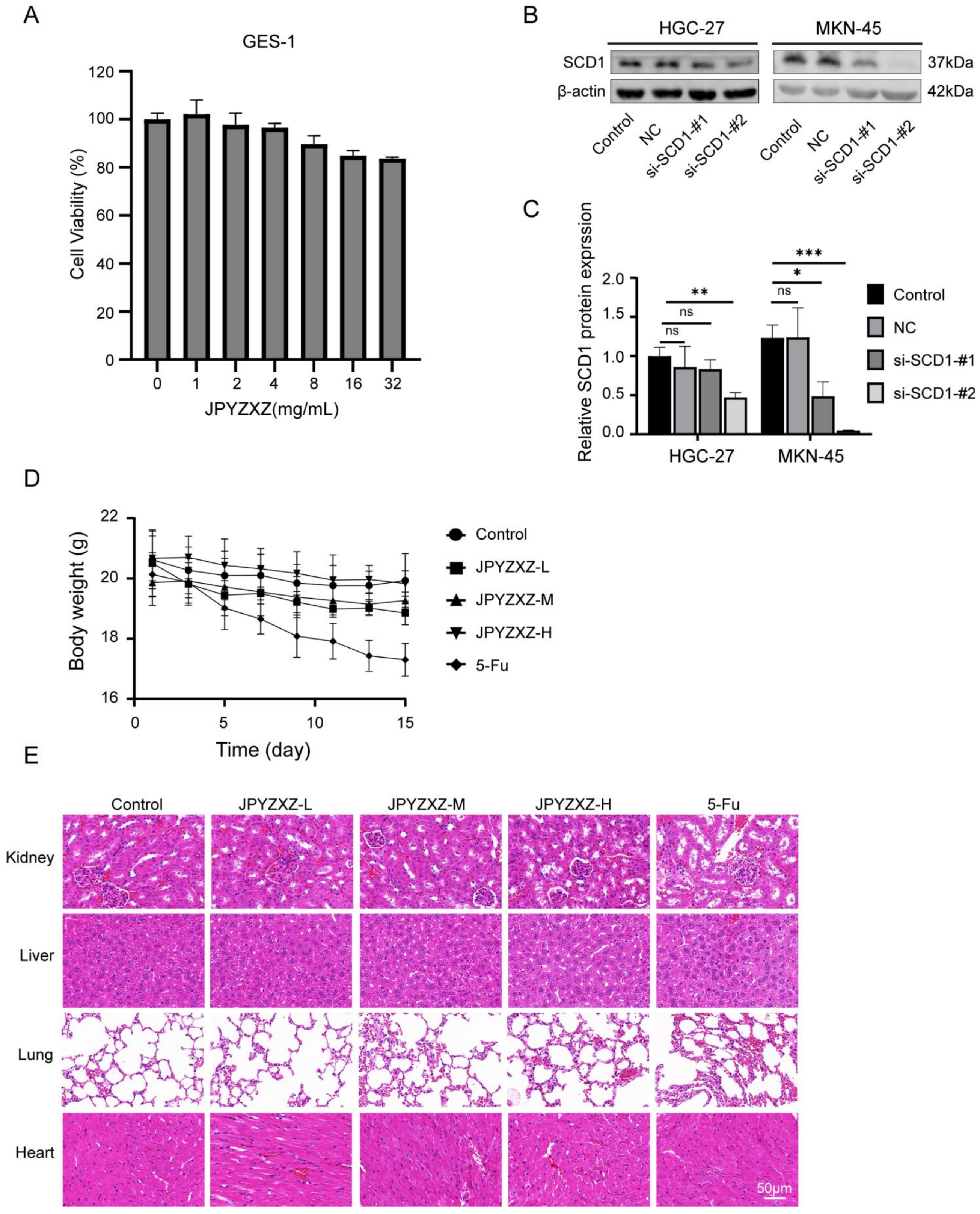

**Supplementary Figure S1.** Safety of JPYXZ in the treatment of gastric cancer in vitro and in vivo. (**A**) The CCK-8 assay was employed to evaluate the toxicity of JPYZXZ on GES-1 cell. (**B**, **C**) The SCD1 knockdown model of GC was verified by Wb assays. (**D**) Fluctuations in body weight over the course of the experiment. (**E**) HE staining was performed on kidney, liver, lung, and heart tissues to assess the safety of JPYZXZ in inhibiting GC xenograft growth. Data are expressed as mean ± SD, n=3. *p<0.05, **p<0.01, ***p<0.001.

**Table S1** The sequence of SCD1

| Lat. No | SCD1 | sequence | |
|---|---|---|---|
| | | sense（5'-3'） | antisense（5'-3'） |
| SR0111D07 | si-SCD1-#1 | GAUAUGCUGUGGUGCUUAATT | UUAAGCACCACAGCAUAUCTT |
| SR0111D09 | si-SCD1-#2 | GCUAGACUUGUCUGACCUATT | UACGUCAGACAAGUCUAGCTT |
